# Supplementary material for: Reinforcement learning establishes a minimal metacognitive process to monitor and control motor learning performance
Source: Nat Commun. 2023 Jul 8;14:3988. doi: 10.1038/s41467-023-39536-9 (PMC10329706; doi:10.1038/s41467-023-39536-9)
Supplement: Supplementary file 1 — Supplementary Information [file 41467_2023_39536_MOESM1_ESM.pdf]

Supplementary Materials

Reinforcement learning establishes a minimal metacognitive process to monitor and control motor learning performance.

Taisei Sugiyama, Nicolas Schweighofer, Jun Izawa

Correspondence to: izawa@emp.tsukuba.ac.jp

Supplementary Figures

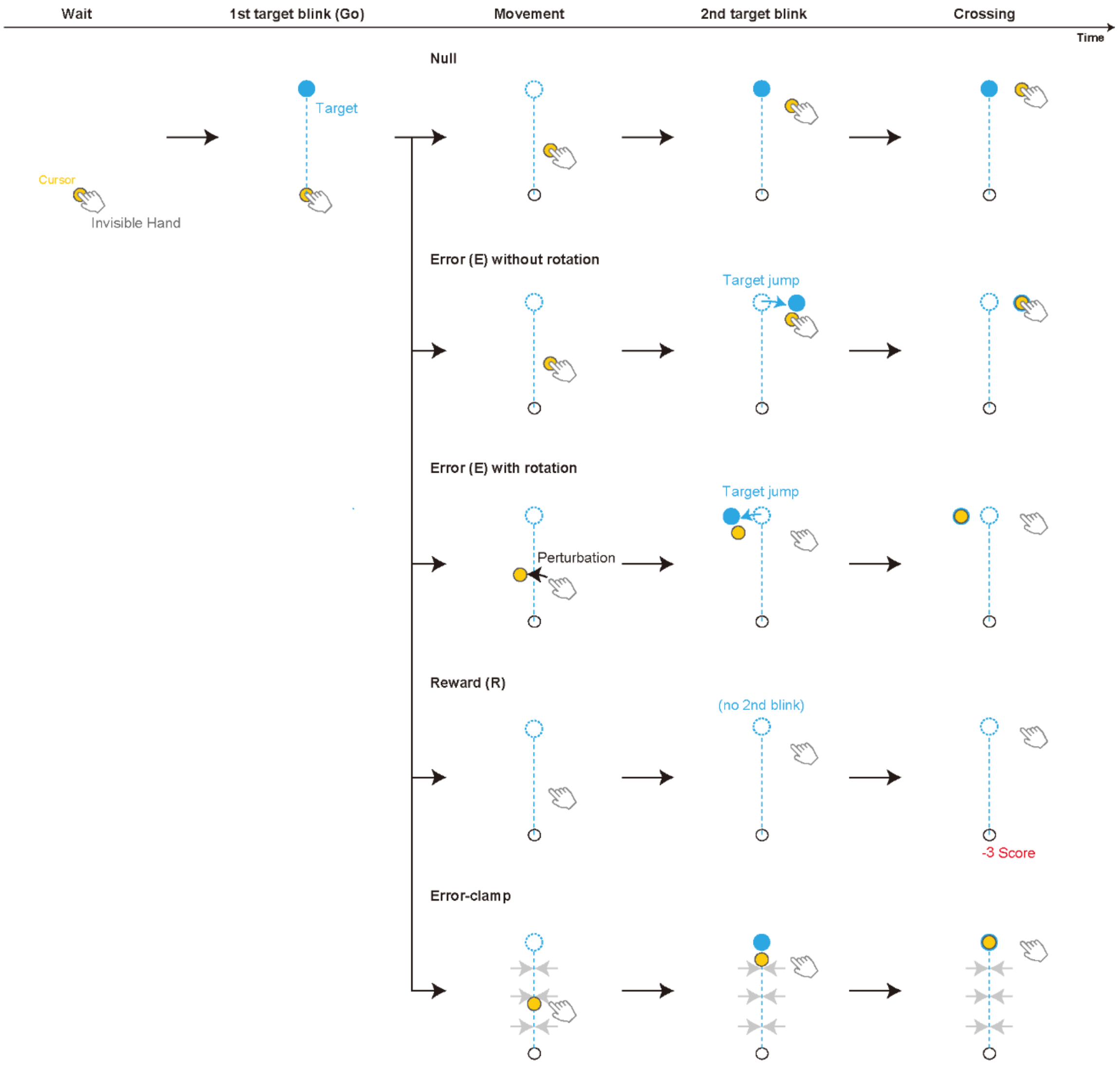

Figure S1.
Flow and manipulation of feedback in the five types of trial. Cursor feedback and second target blink are manipulated differently in each trial type to control sensory prediction error and task error [10]. To illustrate these manipulations, all panels show the same hand direction, which deviates from the target direction. Also the colors and relative sizes of some visual stimuli are modified for illustration purposes. See the "Task design" section in the main manuscript for actual colors and sizes.

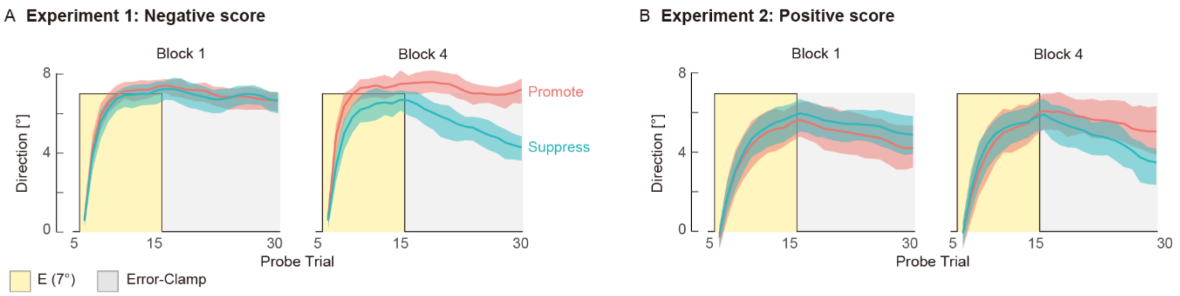

Figure S2.

The posterior predictive distribution with estimated means (solid) and 95% credible intervals (shaded area) of memory profile by the meta-learning model in Blocks 1 and 4 of Experiment 1 with punishment feedback (**A**) and of Experiment 2 with reward feedback (**B**).

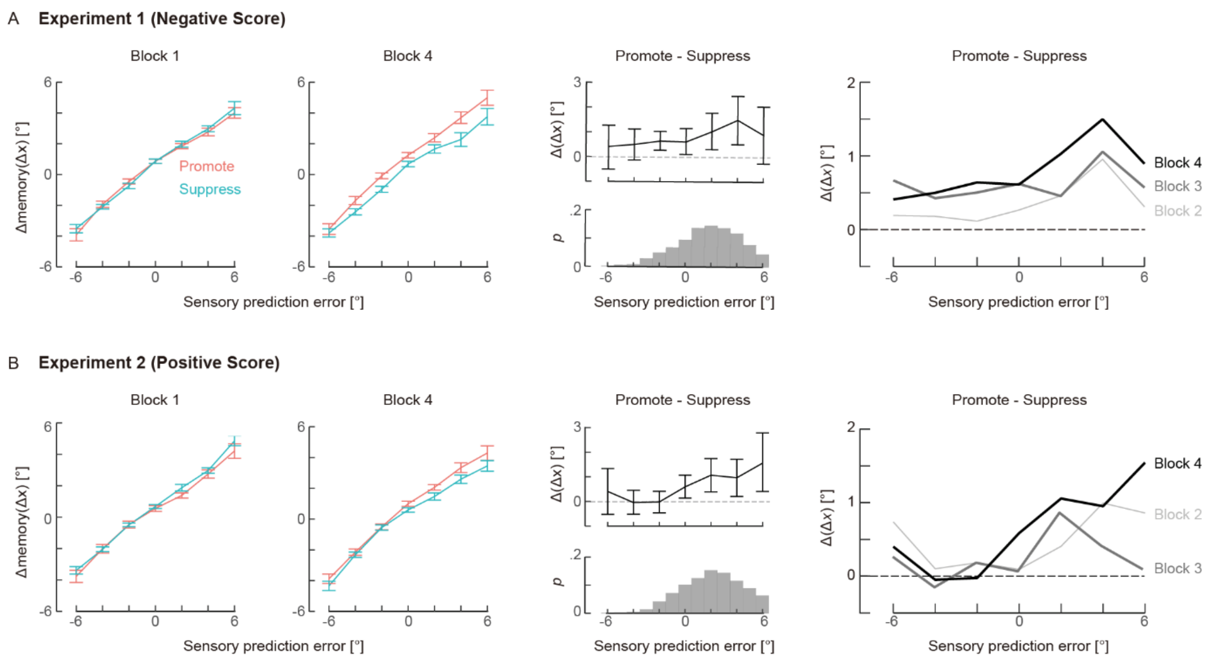

Figure S3.

The effects of meta-learning training on error sensitivity. Error sensitivity during meta-learning training was measured as the average amount of memory update (Δx) over binned sizes of sensory prediction error in the E and null trials. **A.** Experiment 1. Left panel: The error sensitivity of each condition in Block 1 and Block 4. Middle-top panel: Bootstrapped estimates of the group difference ((**Promote**) – (**Suppress**)) in the effects of meta-learning training (Block 4 – Block 1) on the error sensitivity. There is a greater change in the sensitivity ($\Delta(\Delta x)$) on the positive side of the horizontal axis, the sensory prediction error, than on the negative side (p = 0.045). Middle-bottom panel: The probability (p) of the size of the sensory prediction error in the E trial. Right panel: Development of $\Delta(\Delta x)$ over Block, 2, 3 and 4. **B.** Experiment 2. There is a greater change in sensitivity on the positive side than on the negative side of the error space (p = 0.004). Lines represent mean, and error bars represent SEM for observed data (left panels) and 95% confidence interval for bootstrapped data (middle-top). Each mean and SEM are calculated for data from 20 human participants per group (n = 40 per experiment, n = 80 in total).

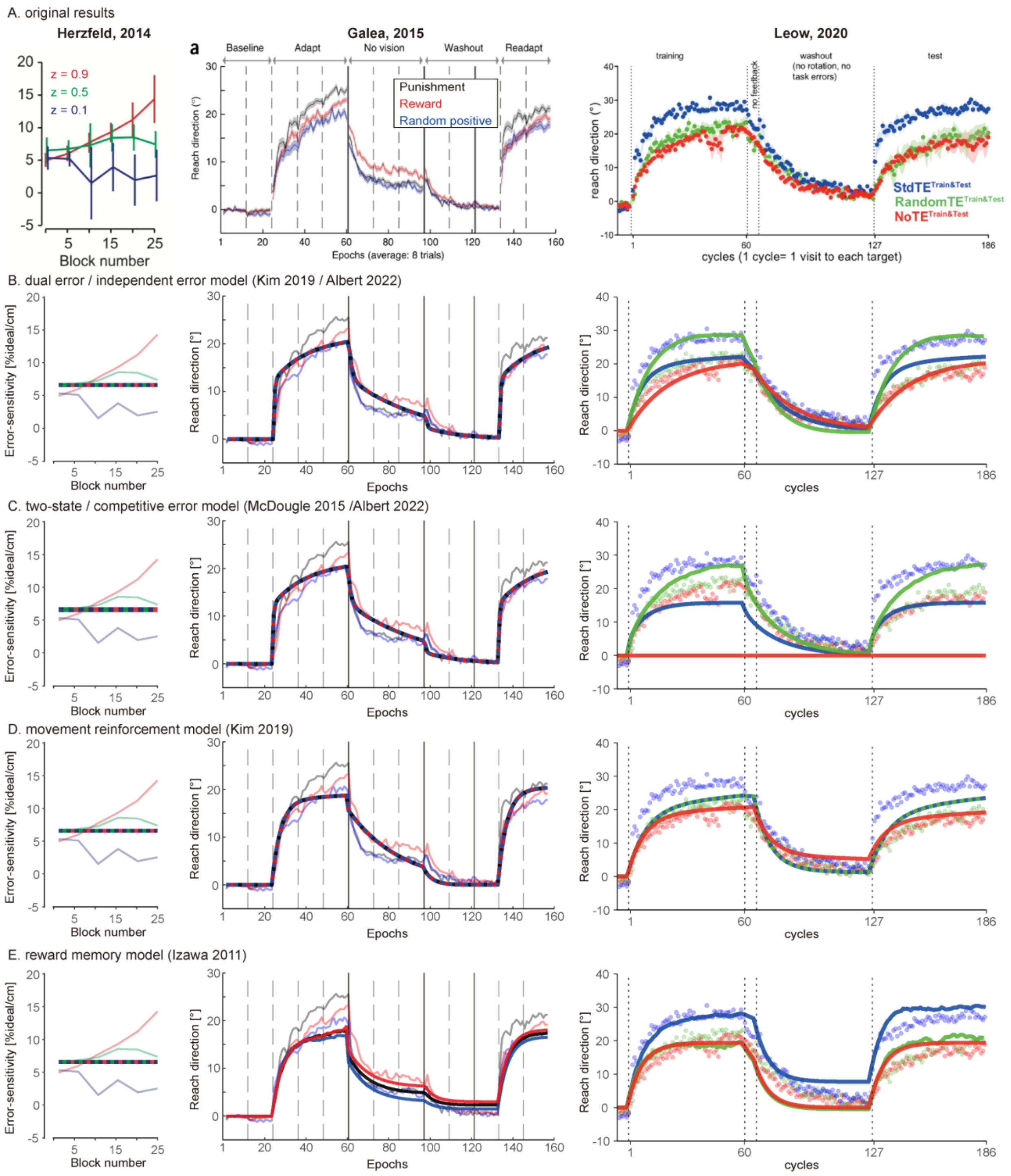

Figure S4.

Simulation results of Herzfeld-2014, Galea-2015, and Leow-2022 with the multi-state models that entail multiple memories. **A.** Original results. **B.** Dual error/independent error model proposed in Kim 2019 and Albert 2022. **C.** Two-state/competitive error models proposed in McDougle 2015

and Albert 2022. **D.** Movement reinforcement model proposed in Kim 2019. **E.** Reward memory model proposed in Izawa 2011. Bold lines indicate simulation fits. Dashed lines are used when multiple lines overlap with each other. Faded lines/dots indicate the mean values from the original studies. For convention, we name some models when they are not in the original studies. In Panel A, the left image is from https://doi.org/10.1126/science.1253138. Reprinted with permission from AAAS. The middle image is adapted with permission from Leow, L. A., Marinovic, W., de Rugy, A., & Carroll, T. J. Task errors drive memories that improve sensorimotor adaptation. J Neurosci 40(15), 3075-3088 (2020). https://doi.org/10.1523/JNEUROSCI.1506-19.2020. https://creativecommons.org/licenses/by/4.0/. The right image is reproduced with permission from Springer Nature. Galea, J., Mallia, E., Rothwell, J. et al. The dissociable effects of punishment and reward on motor learning. Nat Neurosci 18, 597–602 (2015). https://doi.org/10.1038/nn.3956, Springer Nature.

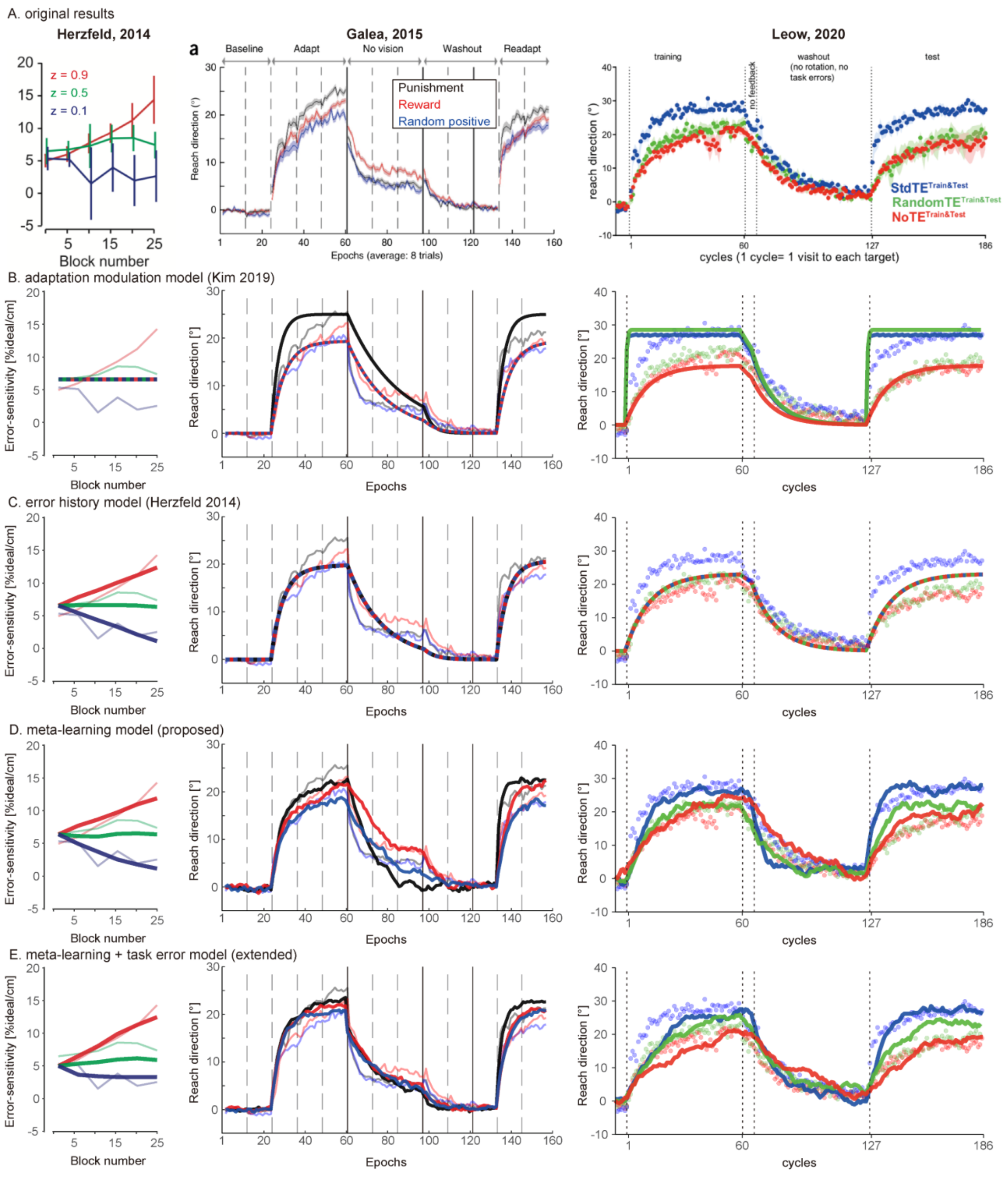

Figure S5.

Simulation results of Herzfeld-2014, Galea-2015, and Leow-2022 with the rate-change models that entail modulation of learning parameters. **A.** Original results. **B.** Adaptation modulation model proposed in Kim 2019. **C.** Error representation model proposed in Herzfeld 2014. **D.** Meta-learning

model proposed in the present study. **E.** Meta-learning + task error model, an extended model in which task error-driven memory is added to the original model. Bold lines indicate simulation fits. Dashed lines are used when multiple lines overlap with each other. Faded lines/dots indicate the mean values from the original studies. For convention, we name some models when they are not in the original studies. In Panel A, the left image is from https://doi.org/10.1126/science.1253138. Reprinted with permission from AAAS. The middle image is adapted with permission from Leow, L. A., Marinovic, W., de Rugy, A., & Carroll, T. J. Task errors drive memories that improve sensorimotor adaptation. J Neurosci 40(15), 3075-3088 (2020). https://doi.org/10.1523/JNEUROSCI.1506-19.2020. https://creativecommons.org/licenses/by/4.0/. The right image is reproduced with permission from Springer Nature. Galea, J., Mallia, E., Rothwell, J. et al. The dissociable effects of punishment and reward on motor learning. Nat Neurosci 18, 597–602 (2015). https://doi.org/10.1038/nn.3956, Springer Nature.

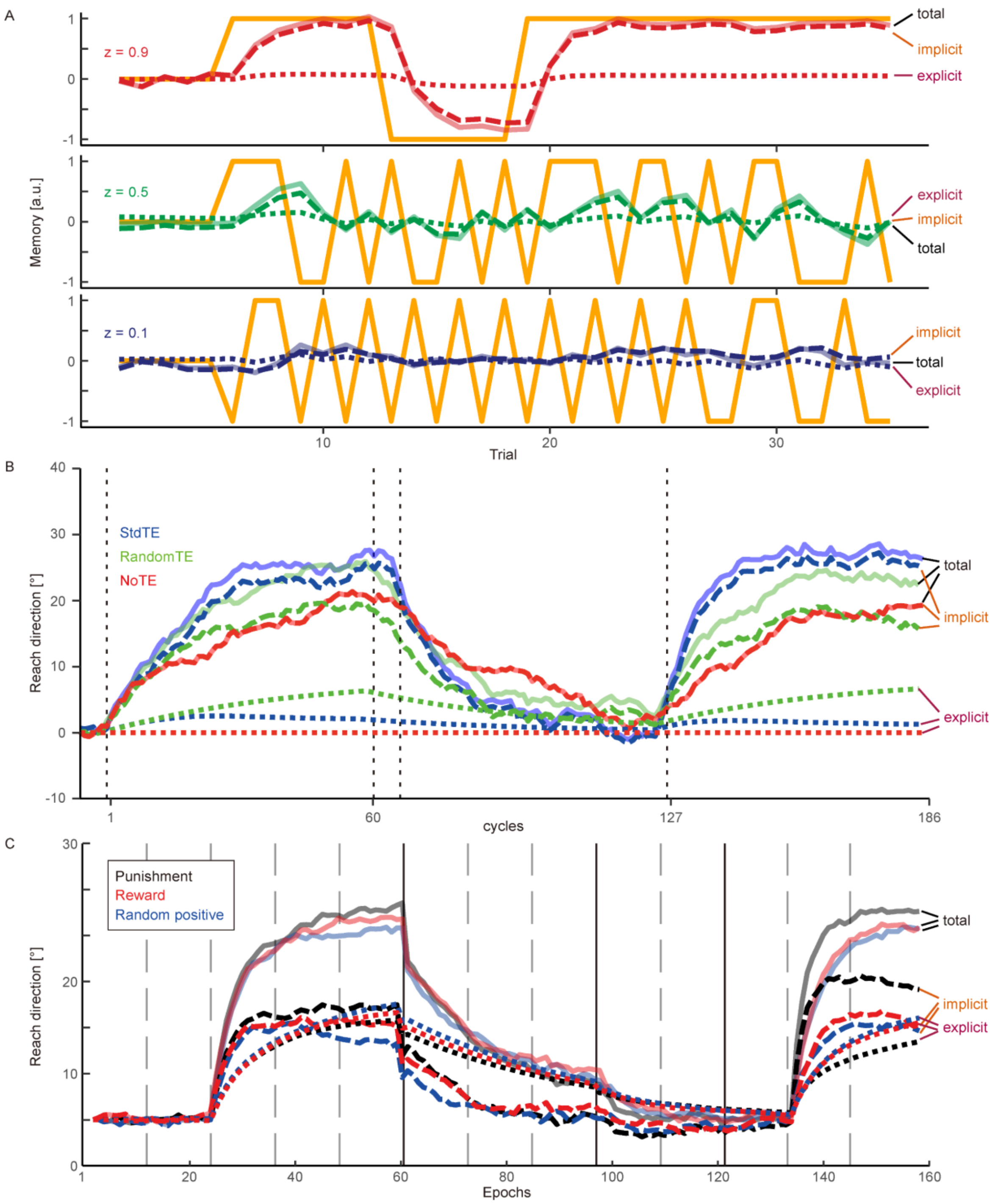

Figure S6.

Total memory that generates observed movements (faded) consists of implicit memory (dashed) and explicit memory (dotted) in the extended meta-learning + target error model. Contributions of implicit memory and explicit memory are determined through optimization of free parameters.

A. Herzfeld-2014. **B.** Leow-2020. **C.** Galea-2015. Lines indicate means. For visualization, standard errors are omitted. a.u. = arbitrary unit.

Supplementary Tables

Equation	$d \sim \gamma_0 + \gamma_1 LOS + \gamma_2 Block + \gamma_3 LOS \cdot Block$				
Group/Data type	Factor	Estimated	Std. Error	*t*-value	*p*
Initial update	γ_0:Intercept	2.04	0.34	6.03	5.0×10^{-8}
	γ_1:LOS	-0.03	0.48	-0.06	.95
	γ_2: Block	-0.11	0.13	-0.84	.40
	**γ_3: LOS·Block**	**0.44**	**0.18**	**2.42**	**.02**
Accumulated update	γ_0:Intercept	3.02	0.38	7.89	5.6×10^{-11}
	γ_1:LOS	0.24	0.54	0.44	.66
	γ_2: Block	-0.40	0.12	-3.39	.001
	**γ_3: LOS·Block**	**0.79**	**0.17**	**4.75**	**5.7×10^{-6}**

Table S1.

Experiment 1. The estimated effect of each factor and interaction in linear mixed model analyses on the Memory size (hand direction) in the initial update and accumulated update in the meta-learning training. As in the main manuscript, residuals and random effects are omitted in the equations for simplicity. The statistical test is two-sided. LOS: Learning-Outcome Structure

Equation	$y \sim \gamma_0 + \gamma_1 Block$				
Group/Data type	Factor	Estimated	Std. Error	t-value	p
Initial update, Promote	γ_0:Intercept	2.00	0.32	6.21	2.1×10^{-7}
	γ_1:Block	0.33	0.12	2.65	.01
Initial update, Suppress	γ_0:Intercept	2.03	0.35	5.77	1.7×10^{-6}
	γ_1:Block	-0.11	0.13	-0.82	.41
Accumulated update, Promote	γ_0:Intercept	3.26	0.38	8.47	1.3×10^{-9}
	γ_1:Block	0.39	0.12	3.31	.002
Accumulated update, Suppress	γ_0:Intercept	3.02	0.34	7.60	2.1×10^{-7}
	γ_1:Block	-0.40	0.12	-3.41	.001

Table S2.

Experiment 1. The estimated effect of each factor on the Memory size (hand direction) in the initial update and accumulated update in the meta-learning training. Each group is separately analyzed to compare its effects with a hypothetical mean of 0. Estimated slopes (the effects of block) are plotted in **Fig. 2B**. The statistical test is two-sided.

Equation	$y \sim \gamma_0 + \gamma_1 LOS + \gamma_2 Block + \gamma_3 LOS \cdot Block$				
Group/Data type	Factor	Estimated	Std. Error	t-value	p
Score	γ_0:Intercept	-2.42	0.22	-10.88	4.4×10^{-14}
	γ_1:LOS	-0.69	0.31	-2.19	0.03
	γ_2: Block	0.22	0.03	6.88	6.6×10^{-12}
	γ_3: LOS·Block	0.04	0.05	0.88	0.38

Table S3.

Experiment 1. The estimated effect of each factor on the Score performance in meta-learning training. The statistical test is two-sided.

Equation	$y \sim \gamma_0 + \gamma_1 Block$				
Group/Data type	Factor	Estimated	Std. Error	*t*-value	*p*
Score, Promote	γ_0:Intercept	-3.11	0.23	-13.40	4.7×10^{-12}
	**γ_1:Block**	**0.27**	**0.03**	**7.95**	**2.9×10^{-15}**
Score, Suppress	γ_0:Intercept	-2.42	0.21	-11.40	9.5×10^{-11}
	**γ_1:Block**	**0.22**	**0.03**	**7.05**	**2.4×10^{-12}**

Table S4.

Experiment 1. The estimated effect of each factor on the Score. Each group is analyzed separately to compare effects with a hypothetical mean of 0. Estimated slopes (the effects of block) are plotted in **Fig. 2C**. The statistical test is two-sided.

Equation	$y \sim \gamma_0 + \gamma_1 LOS + \gamma_2 Block + \gamma_3 LOS \cdot Block$				
Group/Data type	Factor	Estimated	Std. Error	*t*-value	*p*
Initial update	γ_0:Intercept	2.92	0.52	5.65	1.7×10^{-7}
	γ_1:LOS	0.14	0.73	0.19	.85
	γ_2: Block	0.004	0.21	0.02	.99
	**γ_3: LOS·Block**	**0.63**	**0.30**	**2.06**	**.04**
Retention	γ_0:Intercept	6.21	0.47	13.17	2.0×10^{-19}
	γ_1:LOS	-0.14	0.67	-0.22	.83
	γ_2: Block	-0.56	0.14	-4.01	.0001
	**γ_3: LOS·Block**	**0.58**	**0.20**	**2.95**	**.004**

Table S5.

Experiment 1. The estimated effect of each factor on the Memory size (hand direction) in the initial update and retention in the Probe. The statistical test is two-sided.

Equation	$y \sim \gamma_0 + \gamma_1 Block$				
Group/Data type	Factor	Estimated	Std. Error	*t*-value	*p*
Initial update, Promote	γ_0:Intercept	3.05	0.57	5.36	4.7×10^{-6}
	γ_1:Block	0.63	0.20	3.12	.003
Initial update, Suppress	γ_0:Intercept	2.92	0.46	6.37	2.1×10^{-8}
	γ_1:Block	0.004	0.23	0.02	.99
Retention, Promote	γ_0:Intercept	6.06	0.55	11.00	8.7×10^{-12}
	γ_1:Block	0.02	0.15	0.14	.89
Retention, Suppress	γ_0:Intercept	6.20	0.37	16.56	6.4×10^{-18}
	γ_1:Block	-0.56	0.12	-4.45	3.7×10^{-5}

Table S6.

Experiment 1. The estimated effect of each factor on the Memory size (hand direction) in the initial update and retention in the Probe. Each group is separately analyzed to compare effects with a hypothetical mean of 0. Estimated slopes (the effects of block) are plotted in **Fig. 2E**. The statistical test is two-sided.

Equation	$d \sim \gamma_0 + \gamma_1 LOS + \gamma_2 Block + \gamma_3 LOS \cdot Block$				
Group/Data type	Factor	Estimated	Std. Error	*t*-value	*p*
Initial update	γ_0:Intercept	2.03	0.31	6.52	7.1×10^{-9}
	γ_1:LOS	-0.34	0.44	-0.78	.44
	γ_2: Block	-0.04	0.11	-0.37	.71
	**γ_3: LOS·Block**	**0.22**	**0.16**	**1.35**	**.18**
Accumulated update	γ_0:Intercept	3.07	0.35	8.69	2.2×10^{-12}
	γ_1:LOS	0.07	0.50	0.14	.89
	γ_2: Block	-0.18	0.11	-1.67	.10
	**γ_3: LOS·Block**	**0.26**	**0.15**	**1.68**	**.10**

Table S7.

Experiment 2. The estimated effect of each factor on the Memory size (hand direction) in the initial update and accumulated update in meta-learning training. The statistical test is two-sided.

Equation	$d \sim \gamma_0 + \gamma_1 Block$				
Group/Data type	Factor	Estimated	Std. Error	*t*-value	*p*
Initial update, Promote	γ_0:Intercept	1.68	0.36	4.72	3.8×10^{-8}
	γ_1:Block	0.17	0.12	1.46	.15
Initial update, Suppress	γ_0:Intercept	2.03	0.26	7.87	4.3×10^{-10}
	γ_1:Block	-0.04	0.11	-0.40	.69
Accumulated update, Promote	γ_0:Intercept	3.14	0.37	8.49	2.2×10^{-9}
	γ_1:Block	0.08	0.10	0.74	.46
Accumulated update, Suppress	γ_0:Intercept	3.07	0.34	9.14	8.8×10^{-11}
	γ_1:Block	-0.18	0.11	-1.60	.11

Table S8.

Experiment 2. The estimated effect of each factor on the Memory size (hand direction) in the initial update and accumulated update in the meta-learning training. Each group is analyzed separately to compare effects with a hypothetical mean of 0. Estimated slopes (the effects of block) are plotted in Fig. **3B**. The statistical test is two-sided.

Equation	$d \sim \gamma_0 + \gamma_1 LOS + \gamma_2 Block + \gamma_3 LOS \cdot Block$				
Group/Data type	Factor	Estimated	Std. Error	*t*-value	*p*
Score	γ_0:Intercept	7.51	0.21	35.65	1.1×10^{-34}
	γ_1:LOS	-0.96	0.30	-3.21	0.002
	γ_2: Block	0.17	0.03	5.13	3.1×10^{-7}
	γ_3: LOS·Block	**-0.15**	**0.05**	**-3.03**	**0.002**

Table S9.

Experiment 2. The estimated effect of each factor on the Score performance in meta-learning training. The statistical test is two-sided.

Equation	$d \sim \gamma_0 + \gamma_1 Block$				
Group/Data type	Factor	Estimated	Std. Error	*t*-value	*p*
Score, Promote	γ_0:Intercept	6.56	0.24	27.65	1.0×10^{-18}
	**γ_1:Block**	**0.03**	**0.04**	**0.79**	**.43**
Score , Suppress	γ_0:Intercept	7.51	0.18	41.62	3.2×10^{-23}
	**γ_1:Block**	**0.17**	**0.03**	**5.49**	**4.4×10^{-8}**

Table S10.

Experiment 2. The estimated effect of each factor on the Score. Each group is separately analyzed to compare the effects with a hypothetical mean of 0. Estimated slopes (the effects of block) are plotted in Fig. **3C**. The statistical test is two-sided.

Equation	$y \sim \gamma_0 + \gamma_1 LOS + \gamma_2 Block + \gamma_3 LOS \cdot Block$				
Group/Data type	Factor	Estimated	Std. Error	*t*-value	*p*
Initial update	γ_0:Intercept	2.44	0.56	4.39	3.0×10^{-5}
	γ_1:LOS	0.27	0.79	0.34	.73
	γ_2: Block	0.09	0.23	0.38	.71
	**γ_3: LOS·Block**	**0.07**	**0.32**	**0.22**	**.82**
Retention	γ_0:Intercept	6.01	0.47	13.81	9.1×10^{-19}
	γ_1:LOS	-0.50	0.66	-0.75	.46
	γ_2: Block	-0.29	0.14	-2.16	.03
	**γ_3: LOS·Block**	**0.47**	**0.19**	**2.47**	**.01**

Table S11.

Experiment 2. The estimated effect of each factor on the Memory size (hand direction) in the initial update and retention in the Probe. The statistical test is two-sided.

Equation	$y \sim \gamma_0 + \gamma_1 Block$				
Group/Data type	Factor	Estimated	Std. Error	*t*-value	*p*
Initial update, Promote	γ_0:Intercept	2.71	0.64	4.20	1.5×10^{-4}
	**γ_1:Block**	**0.16**	**0.23**	**0.68**	**.50**
Initial update, Suppress	γ_0:Intercept	2.44	0.45	5.42	8.6×10^{-7}
	**γ_1:Block**	**0.09**	**0.23**	**0.39**	**.70**
Retention, Promote	γ_0:Intercept	5.52	0.54	10.27	5.7×10^{-11}
	**γ_1:Block**	**0.18**	**0.14**	**1.27**	**.21**
Retention, Suppress	γ_0:Intercept	6.01	0.39	15.45	7.0×10^{-17}
	**γ_1:Block**	**-0.29**	**0.13**	**-2.28**	**.03**

Table S12.

Experiment 2. The estimated effect of each factor on the Memory size (hand direction) in the initial update and retention in the Probe. Each group is analyzed separately to compare effects with a hypothetical mean of 0. Estimated slopes (the effects of block) are plotted in Fig. **3E**. The statistical test is two-sided.

parameter	Group/Comparison	lower boundary	mean	upper boundary
$\alpha_{[g,base]}$	Promote*	0.979	0.989	0.997
	Suppress*	0.981	0.990	0.999
$\beta_{[g,base]}$	Promote*	0.414	0.488	0.569
	Suppress*	0.362	0.447	0.534
γ_g^{α}	Promote	-5.07×10^{-3}	-4.01×10^{-5}	4.68×10^{-3}
	Suppress*	-1.78×10^{-2}	-1.21×10^{-2}	-6.83×10^{-3}
	**Promote – Suppress***	**5.34×10^{-3}**	**1.20×10^{-2}**	**2.00×10^{-2}**
γ_g^{β}	Promote*	1.13×10^{-2}	5.63×10^{-2}	0.105
	Suppress	-4.04×10^{-2}	-5.97×10^{-3}	2.71×10^{-2}
	**Promote – Suppress***	**7.17×10^{-3}**	**6.23×10^{-2}**	**0.120**

Table S13.

Experiment 1. Means and upper/lower boundaries of 95% HDI of learning parameters estimated by the MCMC method. A parameter was considered significant when the 95% HDI did not include 0, indicated by an asterisk “*” in the “Group/Comparison” column.

parameter	Group/Comparison	lower boundary	mean	upper boundary
$\alpha_{[g,base]}$	Promote*	0.962	0.973	0.984
	Suppress*	0.972	0.982	0.992
$\beta_{[g,base]}$	Promote*	0.173	0.254	0.342
	Suppress*	0.206	0.275	0.341
γ_g^α	Promote	-2.86×10^{-3}	2.14×10^{-3}	7.95×10^{-3}
	Suppress*	-1.35×10^{-2}	-7.75×10^{-3}	-2.21×10^{-3}
	**Promote – Suppress***	$\mathbf{1.90 \times 10^{-3}}$	$\mathbf{9.89 \times 10^{-3}}$	$\mathbf{1.73 \times 10^{-2}}$
γ_g^β	Promote	-1.56×10^{-2}	5.83×10^{-3}	2.68×10^{-2}
	Suppress	-4.83×10^{-3}	1.83×10^{-2}	4.12×10^{-2}
	**Promote - Suppress**	$\mathbf{-4.36 \times 10^{-2}}$	$\mathbf{-1.24 \times 10^{-2}}$	$\mathbf{1.91 \times 10^{-2}}$

Table S14.

Experiment 2. Means and upper/lower boundaries of 95% HDI of learning parameters estimated by the MCMC method. A parameter was considered significant when the 95% HDI did not include 0, indicated by an asterisk “*” in the “Group/Comparison” column.

parameter	Group/Comparison	lower boundary	mean	upper boundary
η_α	**Negative***	**3.84×10^{-6}**	**6.26×10^{-5}**	**1.33×10^{-4}**
	**Positive***	**1.72×10^{-5}**	**1.07×10^{-4}**	**1.96×10^{-4}**
	**Negative – Positive**	**-1.48×10^{-4}**	**-4.39×10^{-5}**	**7.16×10^{-5}**
η_β	**Negative***	**3.65×10^{-5}**	**2.85×10^{-4}**	**5.51×10^{-4}**
	**Positive**	**-1.91×10^{-4}**	**-4.70×10^{-5}**	**9.07×10^{-5}**
	**Negative – Positive***	**3.19×10^{-5}**	**3.32×10^{-4}**	**6.39×10^{-4}**

Table S15.

Experiments 1 and 2. Means and upper/lower boundaries of 95% HDI of the meta-learning rates (η, **Fig. 3G**) estimated by the MCMC method. A parameter was considered significant when the 95% HDI did not include 0, indicated by an asterisk "*" in the "Group/Comparison" column.

	condition	σ_x	$\alpha^{(1\|1)}$	$\beta^{(1\|1)}$	η_α	η_β	C_{TE}	C_S
Herzfeld-2014	z = 0.9	0.1	0.90	0.06	0.4	0.4	n.a.	n.a.
	z = 0.5							
	z = 0.1							
Galea-2015	Punishment	5.0	0.90	0.10	1.2×10^{-8}	2.8×10^{-7}	1.0	20.0
	Reward				6×10^{-8}	0		
	Random-positive				6×10^{-8}	0		
Leow-2020	StdTE	3.0	0.99	0.015	1.1×10^{-8}	4.4×10^{-8}	n.a.	n.a.
	RandomTE							
	NoTE							

Table S16.

Free parameter values for each condition and study for simulation of previous studies. n.a. = not applicable.

	Herzfeld-2014	Galea-2015	Leow-2020
**Multi-state**			
I. dual error /independent (Kim 2019 / Albert 2022)	×	×	×
II. two-state / competitive (McDougle 2015 / Albert 2022)	×	×	×
III. movement reinforcement (Kim 2019)	×	×	×
IV. reward memory (Izawa 2011)	×	×	✓
**Modulation of learning**			
V. adaptation modulation (Kim 2019)	×	×	×
VI. error representation (Herzfeld 2014)	✓	×	×
VII. meta-learning (proposed)	✓	✓	✓
VIII. meta-learning + task error (hybrid)	✓	✓	✓

Table S17.

Qualitative evaluation of various models for Herzfeld-2014, Galea-2015, and Leow-2022. The criteria for the qualitative evaluation are set for each study as following. For Herzfeld-2014, the error sensitivity changes differently across the conditions, increasing, unchanging, and decreasing in stable ($z = .9$), middle ($z = .5$), and unstable ($z = .1$) environments, respectively. For Galea-2015, adaptation is faster in Punishment while retention during no vision is stronger in Reward. Also, Random positive should not show either faster adaptation or stronger retention. For Leow-2020, StdTE shows more adaptation than RandomTE or NoTE while RandomTE and NoTE show a comparable level of adaptation. Checkmarks indicate successful replication, meeting all the

criteria. Orange crosses indicate partial replication, meeting some but not all of the criteria. Red crosses indicate unsuccessful replication, meeting none of the criteria.

	Herzfeld-2014	Galea-2015	Leow-2020
Multi-state			
I. dual error /independent (Kim 2019 / Albert 2022)	3.1%	2.1°	4.8°
II. two-state / competitive (McDougle 2015 / Albert 2022)	3.1%	2.1°	9.7°
III. movement reinforcement (Kim 2019)	3.1%	2.3°	3.7°
IV. reward memory (Izawa 2011)	3.1%	2.4°	**3.0°**
Modulation of learning			
V. adaptation modulation (Kim 2019)	3.1%	3.0°	6.5°
VI. error history (Herzfeld 2014)	**1.3%**	2.4°	4.1°
VII. meta-learning (proposed)	**1.1%**	**1.9°**	**2.8°**
VIII. meta-learning + task error (extended)	**1.1%**	**1.9°**	**3.1°**

Table S18.

Quantitative evaluation of the models for Herzfeld-2014, Galea-2015, and Leow-2022 by the root mean squared error. Small values indicate more accurate fit. Values are bolded for qualitatively successful simulations (**Table S17**). Error is calculated for the error sensitivity (% ideal/cm) for Herzfeld-2014 and hand direction in ° for Galea-2015 and Leow-2020.

	Herzfeld-2014	Galea-2015	Leow-2020
Multi-state			
dual error /independent ($\alpha_{spe}, \alpha_{te}, \beta_{spe}, \beta_{te}$)	(.90, .80, .06, .004)	(.98, 1.00, .05, .54)	(.998, .992, .004, .008)
two-state / competitive ($\alpha_1, \alpha_2, \beta_1, \beta_2$)	(.90, .80, .06, .004)	(.97, 1.00, .05, .55)	(.993, .88, .007, .009)
movement reinforcement ($\alpha, \beta, A'_{jj}, s$)	(.9999, .07, .999, .9.7×10^{-4})	(.96, .17, .996, 3.6×10^{-4})	(.999, .01, .999, .001)
reward memory ($\alpha, \beta, \rho, r_{base}, r_{base-rwd}, \sigma$)	(.91, .07, .03, -3.1, n.a., 1.0)	(.91, .09, .02, -2.02, .06, 2.1)	(.994, .01, .09, 0, n.a., .87)
Rate-change			
adaptation modulation ($\alpha, \beta, \lambda_\alpha, \lambda_\beta$)	(.999, .07, 1.00, .49)	(.96, .24, .99, .47)	(.992, .23, 1.00, .03)
error history ($\alpha^{(1)}, \beta^{(1)}, \zeta$)	(.96, .07, 1.0×10^{-4})	(.94, .10, 2.7×10^{-4})	(.998, .008, 0)
meta-learning ($\alpha^{(1\|1)}, \beta^{(1\|1)}, \eta_{\alpha-pun}, \eta_{\beta-pun}, \eta_{\alpha-rwd}, \eta_{\beta-rwd}, \sigma$)	(.90, .06, .004, .001, n.a., n.a., .18)	(.90, .10, 3.0×10^{-7}, 7.0×10^{-6}, 2.3×10^{-6}, 7.0×10^{-7}, 5.0)	(.999, .004, 2.0×10^{-7}, 6.8×10^{-7}, n.a., n.a., 2.0)
meta-learning +task error ($\alpha^{(1\|1)}, \beta^{(1\|1)}, \alpha_{te}, \beta_{te}, \eta_{\alpha-pun}, \eta_{\beta-pun}, \eta_{\alpha-rwd}, \eta_{\beta-rwd}, \sigma,$)	(.90, .02, .90, .03, .004, .001, n.a., n.a., .2)	(.90, .08, .97, .05, 1.1×10^{-7}, 8.6×10^{-6}, 5.3×10^{-7}, 8.6×10^{-7}, 3.4)	(.999, .003, .997, .001, 1.0×10^{-7}, 4.0×10^{-7}, n.a., n.a., 1.6)

Table S19.

Estimated optimal free parameters for each model and study. n.a. = not applicable.

Supplementary Note 1

Our theory suggests that meta-learning is mediated by the neural policy function for memory update. Ample evidence suggests that the learning system in the brain is formed over sensory primitives with local receptive fields; thus, learning effects exhibit generalization over the experienced sensory space [1-4]. In other words, if the function approximator of the learning system is composed of local basis functions which have receptive fields, a learning experience is generalized only over the activated basis functions. Thus, because the memory update policy is a function of such errors in our theory, we predict that the effect of meta-learning on the memory update amount was also local in the range of the sensory prediction errors during training and thus forms the generalization function of meta-learning that has a peak at the most frequently experienced error range. Alternatively, if the memory update is not a function of the experienced error, but a simple cue-response map, the meta-learning effects should also generalize to non-experienced errors, forming the generalization function that is flat.

To examine this, we computed the amount of memory update Δx over the space of the sensory prediction error e during the meta-learning training (**Fig. S3**). Since the updated memory in response to e in trial k (E trial) is measured in the next trial k+1 (R trial), we have:

$$\Delta x^{(k)} = x^{(k+1)} - x^{(k)} = f(e^{(k)}) . \qquad \text{(S1)}$$

We measured Δx in every E trial together with the next R trial (trials k and k+1, trials k+2 and k+3, and so on).

The group mean Δx was measured in binned regions of e for each condition and block. Precisely, the individual mean Δx was calculated for each binned region using a sliding window with a width of 3°. The value of each bin was plotted at the center of each bin aligned between −6° and 6°. The group mean was calculated from the individual means (**Figs. S3 AB** left panels). In addition, to illustrate the group difference in the effect of meta-learning on error sensitivity, we estimated error sensitivity by bootstrap sampling. Here, the same number of participants in each condition (n = 20) was resampled with replacement, and Δx was calculated for each condition and block using the same sliding window. Then, a change in Δx from Block 1 [$\Delta(\Delta x)$] was measured in each block, and a group difference ((**Promote**) – (**Suppress**)) was calculated. The mean and 95% confidence interval of $\Delta(\Delta x)$ were calculated from 5000 bootstrap samples (**Figs. S3 AB** middle-top panels and right panels). The probability of the size of sensory prediction error in E

trials across Blocks 2-4 was also calculated from both conditions (**Promote** and **Suppress**) combined (**Figs. S3 AB**, middle-bottom panels).

Because the frequency of the experienced error *e* was biased to the positive side of the *e* space (**Figs. S3 AB** middle-bottom panels), we hypothesized that the meta-learning effect was also biased to the positive side of *e* space because of the generalization effect [3, 4] of meta-learning. If this is true, we should see that the change in the sensitivity $\Delta(\Delta x)$ was larger on the positive side of *e* than on the negative side. To examine this, the average $\Delta(\Delta x)$ was calculated for the positive side and negative side of *e* and the difference between them, $\Delta(\Delta x)$ on the negative side minus $\Delta(\Delta x)$ on the positive side, *negative-positive,* was calculated in each bootstrap sample. Since the null hypothesis was *negative- positive* ≥ 0, we computed the proportion of a bootstrap sample that satisfies this null hypothesis: $p(negative - positive \geq 0) = \#\{negative - positive \geq 0\} / Bs$, where *Bs* (=5000) indicates the number of bootstrap samples [5].

Supplementary Note 2

In past years, a number of motor learning models have been proposed to explain a variety of learning profiles[1, 2, 6-8]. Here, we compare these representative models with our meta-learning model by simulating them in the three different experimental settings (volatility in Herzfeld-2014[1], valence in Galea-2015[9], and task error manipulation in Leow-2020[10]) (**Fig 4**). We compare the models both qualitatively by categorical criteria for successful replication (**Table S17**) and quantitatively by the root mean squared error (RMSE, **Table S18**).

There are three specific aims for this model comparison. First, we show that the previous models can account for only a subset of the datasets of Herzfeld-2014, Gala-2015, and Leow-2020. Second, we demonstrate that our meta-learning model, where the higher reinforcement learning trains the lower motor learning, is the only model that can explain the diverse learning in these three previous studies in a unified manner. Third, we show that the recently established idea of the task error as a driving input to update motor memory does not interfere with the meta-learning model but instead may coexist with the meta-learning. By this model comparison, we show that our meta-learning model is dissociable from previous models not only conceptually (e.g., meta-cognitive structure, hierarchical structure, and optimization principle) but also in terms of explanation ability.

To organize these comparisons, we categorized these models into two classes. One is the "multi-state models" where two memory states are updated by different driving sources and the motor outputs are generated as a sum of these two states[6-8]. In this category, the learning parameters (learning speed and/or retention rate) are constant. The other is called "rate-change models," where some elements alter the learning parameters for updating a single memory state[1, 7].

We apply the nonlinear optimization routine with *nloptr* package[11] in R to estimate optimal free parameter values that minimize fitting errors (RMSE). These estimated values were summarized in **Table S19**. Our proposed meta-learning model and the reward-based learning model[2] are stochastic (i.e., results depend partly on random exploration), and thus the average learning profiles are obtained from multiple simulation runs. We use the same number of runs as the main manuscript for each study (10 for Herzfeld-2014, 200 for Galea-2015, and 100 for Leow-2020). The other models[1, 6-8] are deterministic (i.e., they always produce same results with a given set of parameters), and thus learning profiles are obtained from one simulation run in these models.

For Herzfeld-2014, as the same as the main manuscript (Fig. 1D of Herzfeld-2014), we predicted the experimentally measured error sensitivity $\delta_{ex} = \mu^{(k+1)} / e^{(k)}$ where $\mu^{(k)}$ (a.u.) is the motor memory and $e^{(k)}$ is the sensory error [cm], by using the simulated motor memories. For Galea-2015 and Leow-2020, we simulated the reach angle profiles $h^{(k)}$ over trial and fit parameters of motor learning models to minimize the error between the simulated reach direction profiles and the reported reach direction profiles.

Additional, model-specific assumptions are discussed in each of the sections below. For these model fittings, we extracted group mean behavior values from the plots in these three papers by WebPlotDigitizer (https://apps.automeris.io/wpd/) [12].

Multi-state models

Models in this category entail more than one state (memory), which are summed up to form a total memory. These states are updated by different inputs, such as sensory prediction error, task error, and reward feedback. There are four categories of model in this class.

(I) dual error / independent error model (Kim 2019[7], Albert 2022[8]).

This family of models assumes two different states with two different types of error, sensory prediction error (*spe*) and task (target) error (*te*), which are summed up to form a total state, as following:

$$
\begin{aligned}
x_{spe}^{(k+1)} &= \alpha_{spe} x_{spe}^{(k)} + \beta_{spe} e_{spe}^{(k)}, \\
x_{te}^{(k+1)} &= \alpha_{te} x_{te}^{(k)} + \beta_{te} e_{te}^{(k)}, \\
x_{total}^{(k)} &= x_{spe}^{(k)} + x_{te}^{(k)},
\end{aligned}
\tag{S2}
$$

where $x_{spe}^{(k)}, x_{te}^{(k)}$ are the memory on trial *k* driven by the sensory prediction error and the task error respectively. The motor output is determined by the total memory $x_{total}^{(k)}$: For Herzfels-2014, the memory is $\mu^{(k)} = x_{total}^{(k)}$ and for Galea-2015 and Leow-2020, the reach angle shift is $h^{(k)} = x_{total}^{(k)}$.

Figure S4B shows the simulation results for these models. Since this family does not assume the change in the learning parameters ($\alpha_{spe}, \beta_{spe}, \alpha_{te}, \beta_{te}$) by experience or valence (reward or punishment), it cannot account for Herzfeld-2014 or Galea-2015. In Leow-2020, the task error e_{te} is randomly clamped to 20-30° in RandomTE condition, which is larger than e_{te} in StdTE condition when adaptation progresses. Therefore, the simulation result for RandomTE shows the largest overall adaptation, which contradicts with the results reported in the original paper (Leow-2020). Thus, this family fails to replicate any of the three studies.

(II) two-state / competitive error model (McDougle 2015[6] / Albert 2022[8])

This family of models is identical to (I) except that the same error updates two different states, as following:

$$
\begin{aligned}
x_1^{(k+1)} &= \alpha_1 x_1^{(k)} + \beta_1 e^{(k)}, \\
x_2^{(k+1)} &= \alpha_2 x_2^{(k)} + \beta_2 e^{(k)}, \\
x_{total}^{(k)} &= x_1^{(k)} + x_2^{(k)},
\end{aligned}
\tag{S3}
$$

where $e^{(k)}$ is defined as task error in Albert 2022, whereas it is simply called "error" in McDougle 2015 because they do not dissociate errors. Also, in McDoungle-2015, x_1 and x_2 represent fast and slow memories, which adds constraints such that $\alpha_2 < \alpha_1$ and $\beta_1 < \beta_2$. Similarly to the model family (I), the motor output is determined by the total memory $x_{total}^{(k)}$: For Herzfeld-2014, the

generated compensation force is $\mu^{(k)} = x_{total}^{(k)}$ and for Galea-2015 and Leow-2020, the reach angle shift is $h^{(k)} = x_{total}^{(k)}$.

Figure S4C shows the simulation results. As in (I), it cannot account for change in the learning parameters ($\alpha_1, \beta_1, \alpha_2, \beta_2$) by experience or valence in Herzfeld-2014 or Galea-2015. Also, in Leow 2020, RandomTE shows the largest overall adaptation, and NoTE shows no adaptation because *e* is clamped to 0. They both contradict with the original results. Thus, this family also fails to replicate any of the three studies. Note that the constraints for the fast and slow memories are not considered because this family cannot replicate the original results even without the constraints.

(III) movement reinforcement model (Kim 2019[7])

This model assumes that a total memory consists of motor memory *x* and reward memory V_d, which together produces motor output *y*. For update of *x*, they simplified a conventional state-space model because error was clamped in their study. Since it is not the case in the three studies to be simulated, we use a conventional update equation as following:

$$x^{(k+1)} = \alpha x^{(k)} + \beta e_{spe}^{(k)}. \tag{S4}$$

The reward memory V_d follows model-free update rule defined as following:

$$\begin{aligned} V_x^{(k)} &= \mathbf{r}^{(k)} \cdot \mathbf{u}_x, \\ V_y^{(k)} &= \mathbf{r}^{(k)} \cdot \mathbf{u}_y, \\ V_d^{(k)} &= \tan^{-1}(V_y^{(k)} / V_x^{(k)}), \end{aligned} \tag{S5}$$

where ***u*** is a unit vector pointing in different directions and ***r*** is a weight vector updated differently for the rewarded direction θ and other directions ~θ as following:

$$\begin{aligned} \mathbf{r}_{\theta}^{(k+1)} &= A'\mathbf{r}_{\theta}^{(k)} + s, \\ \mathbf{r}_{\sim\theta}^{(k+1)} &= A'\mathbf{r}_{\sim\theta}^{(k)}. \end{aligned} \tag{S6}$$

Then, *x* and V_d are summed with a weight factor V_1 that is computed from V_d as following:

$$y^{(k)} = (1 - V_1^{(k)}) x^{(k)} + V_1^{(k)} V_d^{(k)}, \tag{S7}$$

where $V_1^{(k)} = \sqrt{(V_x^{(k)})^2 + (V_y^{(k)})^2}$. The motor output is determined by $y^{(k)}$. For Herzfeld-2014, the memory is $\mu^{(k)} = y^{(k)}$ and for Galea-2015 and Leow-2020, the reach angle shift is $h^{(k)} = y^{(k)}$.

Figure S4D shows the simulation results. As in (I) and (II), the model cannot account for change in the learning parameters (α and β) by experience in Herzfeld-2014, showing unchanging error sensitivity across the conditions. In this model, contribution of motor memory (x) to an output (y) becomes smaller as reward memory develops, represented by the weighting factor (V_1). Therefore, this model only predicts decreasing error sensitivity over time, which contradicts with the reported increasing error sensitivity in the stable environment (z = 0.9) in Herzfeld-2014. Thus, optimal parameters are found to favor no changes over decreasing error sensitivity across conditions. The model also fails to simulate the effect of valence in Galea-2015. In Leow-2020, NoTE condition leads to consistent target hit (reward) from the beginning, reinforcing the baseline reach direction. As a results, it shows attenuated adaptation compared to StdTE and RandomTE. However, it does not predict more adaptation for StdTE than RandomTE. Thus, this model also fails to replicate any of the three studies.

(IV) reward memory model (Izawa 2011[2])

The model of category (III) treats reward feedback as binary, which makes it difficult to apply to the three experiments where reward feedback is continuous, or where the target hit/miss is not manipulated, as in Kim 2019. Thus, we examine this alternative model in which total memory $y^{(k)}$ also consists of motor memory $x_{spe}^{(k)}$ and reward memory $x_r^{(k)}$. Unlike (III), reward memory is a scalar that is updated by reward prediction error. Therefore, although reward feedback is also binary in the original study, the model can be extended such that update of reward memory x_r is represented with a continuous variable instead of a binary variable (target hit/miss). For simplicity, we use the same conventional update for motor memory used in (I)-(III) and a Rescorla-Wagner update rule with a constant baseline estimation of reward r_{base} with an exploration that follows a normal distribution with constant variance:

$$
\begin{aligned}
x_{spe}^{(k+1)} &= \alpha x_{spe}{}^{(k)} + \beta e_{spe}^{(k)}, \\
x_r^{(k+1)} &= x_r^{(k)} + \rho(r^{(k)} - r_{base}) n^{(k)}, \\
y^{(k)} &= x_{spe}^{(k)} + x_r^{(k)},
\end{aligned}
\tag{S8}
$$

where e_{spe} is the sensory prediction error, ρ is the learning rate of the reinforcement learning, $n^{(k)}$ is the exploration noise. To apply this model to all of the studies, we use the same assumption with the same free parameter values as our main manuscript that target error serves as reward (punishment) signal, defining r as $r^{(k)} = -|e_{te}^{(k)}|$ for Herzfeld-2014 and Leow-2020 and $r^{(k)} = -c_{TE} \cdot |e_{te}^{(k)}| + c_s \cdot score^{(k)}$ for Galea-2015, where e_{te} is the task error, c_{TE}, c_s are weighting factors. Also, for Galea-2015, baseline expected reward was estimated separately for Punishment (r_{base}) and Reward/Random positive ($r_{base\text{-}rwd}$). For Herzfeld-2014, the memory is $\mu^{(k)} = y^{(k)}$ and for Galea-2015 and Leow-2020, the reach angle shift is $h^{(k)} = y^{(k)}$.

Figure **S4E** shows the simulation results. This model does not predict changes in learning rate through experience, failing to replicate Herzfeld-2014. For Galea-2015, it successfully replicates more adaptation and retention in Reward and Punishment than in Random positive (i.e., random reward feedback) condition, but it fails to predict the effect of valence. Therefore, replication is partial. Meanwhile, this model can replicate Leow-2020 because only StdErr condition provides more reward (i.e., less task error) with more adaptation, while reward is independent of adaptation in the other two conditions. Thus, this model successfully replicates one out of the three studies.

Rate-change models

Models in this category entail modulation of learning parameters (retention and/or speed) by experienced sensory prediction error, reward feedback, or both.

(V) adaptation modulation model (Kim 2019[7])

This model assumes modulation parameters $\lambda_\alpha, \lambda_\beta$ in the sensory prediction error $e_{spe}^{(k)}$ driven update of motor memory $x^{(k+1)} = \lambda_\alpha \cdot \alpha x^{(k)} + \lambda_\beta \cdot \beta e_{spe}^{(k)}$. The modulation parameters were set to $\{\lambda_\alpha, \lambda_\beta\} = \{1,1\}$ on miss trials and to optimized parameter values on hit trials, as in Kim 2019. The motor output is determined by the single memory $x^{(k)}$. For Herzfeld-2014, the memory is $\mu^{(k)} = x^{(k)}$ and for Galea-2015 and Leow-2020, the reach angle shift is $h^{(k)} = x^{(k)}$. For Galea-2015, the main finding is dissociable effects between reward and punishment. Therefore, instead of using actual target hit/miss (which obviously leads to the same behavior across the conditions), we treat

Reward and Random positive conditions as target hit and Punishment condition as target miss. For Herzfeld-2014, from the average hand trajectory (available in their manuscript), we assume that their participants mostly miss the target in probe trials, and thus we set $\{\lambda_\alpha, \lambda_\beta\} = \{1,1\}$.

Figure S5B shows the simulation results. This model has no capability to predict change in β through experience in Herzfeld-2014. For Galea-2015, optimization leads to slower learning ($\lambda_\beta < 1$) and more retention ($\lambda_\alpha > 1$) with reward than punishment, which successfully replicates faster adaptation in Punishment and more retention in Reward. However, it fails to account for the differences between Reward and Random positive, and thus replication is partial. For Leow-2020, the estimated optimal λ_α and λ_β are both found to be < 1, likely to simulate less learning with constant target hit in NoTE compared to StdTE. However, this leads to more learning of RandomTE than StdTE because StdTE eventually starts hitting the target, which contradicts the original results. Thus, this model does not replicate any of the three studies either.

(VI) error history model (Herzfeld 2014[1])

This model assumes changes in learning rate β according to history of errors such that β increases or decreases when the direction (sign) of error is consistent or flipped, respectively. Thus, the update rules for motor memory $x^{(k)}$ and β are defined as following:

$$\begin{aligned} x^{(k+1)} &= \alpha x^{(k)} + \beta^{(k)} e_{spe}^{(k)}, \\ \beta^{(k+1)} &= \beta^{(k)} + \zeta \cdot \mathrm{sgn}(e_{spe}^{(k-1)} e_{spe}^{(k)}), \end{aligned} \tag{S9}$$

where α is the retention factor, $e_{spe}^{(k)}$ is the sensory prediction error, ζ is the learning rate of β. While the original model assumes a neural population vector, for simplicity, we reduce the dimension and treat it as a scalar (i.e., a "single neuron") without loss of generality, as in our meta-learning model discussed in the main manuscript. For the replication of Herzfeld-2014, the generated compensation force is $\mu^{(k)} = x^{(k)}$ and for Galea-2015 and Leow-2020, the reach angle shift is $h^{(k)} = x^{(k)}$.

Figure S5C shows the simulation results. This model obviously replicates its own results, Herzfeld-2014. However, it does not replicate Galea-2015 or Leow-2020 because all conditions

experience similar history of sensory prediction errors. Thus, it only replicates one out of the three studies.

(VII) meta-learning model (proposed)

This model is proposed in the present manuscript, which successfully replicate all the three experiments (**Fig. S5D**). Refer to the main manuscript for the detail of formulation.

(VIII) meta-learning + task error model (proposed and hybrid models)

This model is an extension of the proposed model in which task error-driven memory is added and summed with sensory error-driven memory to form a total memory, as in (I). We developed this model to examine whether or not the proposed meta-learning and recent evidence about target error-driven learning interfere with each other.

As in the model category (I) (the dual error/ independent error model), the memory $x_{spe}^{(k)}$ updated by the sensory prediction error $e_{spe}^{(k)}$ and the memory $x_{te}^{(k)}$ updated by the task error $e_{te}^{(k)}$ were added to generate the total memory $x_{total}^{(k)}$, as following,

$$\begin{aligned} x_{spe}^{(k+1)} &= \alpha_{spe}^{(k)} x_{spe}^{(k)} + \beta_{spe}^{(k)} e_{spe}^{(k)}, \\ x_{te}^{(k+1)} &= \alpha_{te} x_{te}^{(k)} + \beta_{te} e_{te}^{(k)}, \\ x_{total}^{(k)} &= x_{spe}^{(k)} + x_{spe}^{(k)}. \end{aligned} \tag{S10}$$

As is the proposed model in the main manuscript, the reinforcement learning updates the learning rates of the sensory prediction error-driven learning policy ($\alpha_{spe}^{(k)}, \beta_{spe}^{(k)}$). Since the reinforcement learning of (α_{te}, β_{te}) has not been examined experimentally, we kept (α_{te}, β_{te}) constant in this simulation, for simplicity. However, the update of (α_{te}, β_{te}) by a cognitive strategy may not deteriorate the explanation ability of this model as long as it is designed optimaly. For the replication of Herzfeld-2014, the generated compensation force is $\mu^{(k)} = x_{total}^{(k)}$ and for Galea-2015 and Leow-2020, the reach angle shift is $h^{(k)} = x_{total}^{(k)}$

Figure S5E shows the simulation results. This extension retains the capability of the meta-learning model to replicate the original studies. Thus, the meta-learning hypothesis does not reject/interfere with recent evidence about differential role of task error-driven learning from

sensory error-driven learning. See also **Figure S6** for contributions of implicit and explicit (task error-driven) memory in each simulation.

Supplementary References

1. Herzfeld, D.J., Vaswani, P.A., Marko, M.K. & Shadmehr, R. A memory of errors in sensorimotor learning. *Science* **345**, 1349-1353 (2014).
2. Izawa, J. & Shadmehr, R. Learning from sensory and reward prediction errors during motor adaptation. *PLoS Comput Biol* **7**, e1002012 (2011).
3. Poggio, T. & Bizzi, E. Generalization in vision and motor control. *Nature* **431**, 768-774 (2004).
4. Donchin, O., Francis, J.T. & Shadmehr, R. Quantifying generalization from trial-by-trial behavior of adaptive systems that learn with basis functions: theory and experiments in human motor control. *J Neurosci* **23**, 9032-9045 (2003).
5. Efron, B. & Tibshirani, R.J. *An introduction to the bootstrap* (CRC press, 1994).
6. McDougle, S.D., Bond, K.M. & Taylor, J.A. Explicit and Implicit Processes Constitute the Fast and Slow Processes of Sensorimotor Learning. *J Neurosci* **35**, 9568-9579 (2015).
7. Kim, H.E., Parvin, D.E. & Ivry, R.B. The influence of task outcome on implicit motor learning. *Elife* **8** (2019).
8. Albert, S.T.*, et al.* Competition between parallel sensorimotor learning systems. *Elife* **11** (2022).
9. Galea, J.M., Mallia, E., Rothwell, J. & Diedrichsen, J. The dissociable effects of punishment and reward on motor learning. *Nat Neurosci* **18**, 597-602 (2015).
10. Leow, L.A., Marinovic, W., de Rugy, A. & Carroll, T.J. Task Errors Drive Memories That Improve Sensorimotor Adaptation. *J Neurosci* **40**, 3075-3088 (2020).
11. Ypma, J., Borchers, H.W. & Eddelbuettel, D. nloptr: R interface to NLopt, 2014. *URL https://CRAN. R-project. org/package= nloptr. R package version* **1**, 12 (2017).
12. Rohatgi, A. Webplotdigitizer: Version 4.6. (2022).
