# Supplementary material for: Reinforcement learning establishes a minimal metacognitive process to monitor and control motor learning performance
Source: Nat Commun. 2023 Jul 8;14:3988. doi: 10.1038/s41467-023-39536-9 (PMC10329706; doi:10.1038/s41467-023-39536-9)
Supplement: Supplementary file 3 — Reporting Summary [file 41467_2023_39536_MOESM3_ESM.pdf]

nature portfolio

Corresponding author(s): Jun Izawa

Last updated by author(s): May 27, 2023

Reporting Summary

Nature Portfolio wishes to improve the reproducibility of the work that we publish. This form provides structure for consistency and transparency in reporting. For further information on Nature Portfolio policies, see our Editorial Policies and the Editorial Policy Checklist.

Statistics

For all statistical analyses, confirm that the following items are present in the figure legend, table legend, main text, or Methods section.

n/a	Confirmed	
☐	☒	The exact sample size (*n*) for each experimental group/condition, given as a discrete number and unit of measurement
☐	☒	A statement on whether measurements were taken from distinct samples or whether the same sample was measured repeatedly
☐	☒	The statistical test(s) used AND whether they are one- or two-sided<br>*Only common tests should be described solely by name; describe more complex techniques in the Methods section.*
☒	☐	A description of all covariates tested
☐	☒	A description of any assumptions or corrections, such as tests of normality and adjustment for multiple comparisons
☐	☒	A full description of the statistical parameters including central tendency (e.g. means) or other basic estimates (e.g. regression coefficient) AND variation (e.g. standard deviation) or associated estimates of uncertainty (e.g. confidence intervals)
☐	☒	For null hypothesis testing, the test statistic (e.g. *F*, *t*, *r*) with confidence intervals, effect sizes, degrees of freedom and *P* value noted<br>*Give P values as exact values whenever suitable.*
☐	☒	For Bayesian analysis, information on the choice of priors and Markov chain Monte Carlo settings
☒	☐	For hierarchical and complex designs, identification of the appropriate level for tests and full reporting of outcomes
☒	☐	Estimates of effect sizes (e.g. Cohen's *d*, Pearson's *r*), indicating how they were calculated

Our web collection on statistics for biologists contains articles on many of the points above.

Software and code

Policy information about availability of computer code

Data collection	Custom codes written in Python 3.7.9 and LabVIEW 2019 for a self-made robot manipulandum
Data analysis	R 4.0.2 with lm, lme4, lmerTest, margins, and cmdstanr packages

For manuscripts utilizing custom algorithms or software that are central to the research but not yet described in published literature, software must be made available to editors and reviewers. We strongly encourage code deposition in a community repository (e.g. GitHub). See the Nature Portfolio guidelines for submitting code & software for further information.

Data

Policy information about availability of data

All manuscripts must include a data availability statement. This statement should provide the following information, where applicable:

- Accession codes, unique identifiers, or web links for publicly available datasets
- A description of any restrictions on data availability
- For clinical datasets or third party data, please ensure that the statement adheres to our policy

Source data are available as an Excel document through Figshare (See the manuscript for the link).

Field-specific reporting

Please select the one below that is the best fit for your research. If you are not sure, read the appropriate sections before making your selection.

☐ Life sciences ☒ Behavioural & social sciences ☐ Ecological, evolutionary & environmental sciences

For a reference copy of the document with all sections, see nature.com/documents/nr-reporting-summary-flat.pdf

Behavioural & social sciences study design

All studies must disclose on these points even when the disclosure is negative.

Study description	Study of human motor behavior with arm movements that are quantitatively measured by a robotic manipulandum.
Research sample	Online advertisement and flyers are used to recruit representative healthy participants in University of Tsukuba. To measure stable motor behavior, recruitment is done with the following conditions. Participants must be age between 18 and 35 and right-handed. They must also report no history of neurological or motor diseases and be able to perform reaching movements at the time of participation. 80 healthy participants volunteered (44 males, age 18-31 years, mean age = 21). Gender differences in the basic ability of visuomotor adaptation are not known, and thus gender-based analyses/adjustments are uncommon in motor adaptation research unless gender itself is a focus of research. Therefore, similar numbers of male and female participants were recruited (26 out of 40 participants and 18 out of 40 were males in Experiment 1 and 2, respectively) to consider and minimize potential gender bias, but gender-based analyses were not conducted.
Sampling strategy	Participants were randomly sampled and assigned to one of the experiment conditions. The sample size per condition in the present study was determined based on sample sizes in recent motor studies with between-participant designs and comparisons (e.g., Lerner 2020, Cerebral Cortex; Codol 2018, Scientific Reports)
Data collection	Data were collected in behavioral experiment sessions with a robotic manipulandum system operated by the experimenter. Each participant was individually scheduled and invited to an experiment session, and therefore only the experimenter and one participant were present at a time. In the motor task, kinematic data were recorded at 1000Hz sampling rate and saved as a text file in each trial of arm-reaching. Saved text file were organized and tagged by a participant number and a trial number. Data collection was complete once a participant finished the last trial, and data processing were performed after that. The experimenter was not blind to the hypotheses of the study.
Timing	Data were collected from August 2021 to February 2022
Data exclusions	No data were excluded from the analyses.
Non-participation	No participant dropped out or declined to participate.
Randomization	Each participant was pseudo-randomly assigned to one of the experiment conditions.

Reporting for specific materials, systems and methods

We require information from authors about some types of materials, experimental systems and methods used in many studies. Here, indicate whether each material, system or method listed is relevant to your study. If you are not sure if a list item applies to your research, read the appropriate section before selecting a response.

Materials & experimental systems

n/a	Involved in the study	
☒	☐	Antibodies
☒	☐	Eukaryotic cell lines
☒	☐	Palaeontology and archaeology
☒	☐	Animals and other organisms
☐	☒	Human research participants
☒	☐	Clinical data
☒	☐	Dual use research of concern

Methods

n/a	Involved in the study	
☒	☐	ChIP-seq
☒	☐	Flow cytometry
☒	☐	MRI-based neuroimaging

Human research participants

Policy information about studies involving human research participants

Population characteristics	See "Research sample" section above
Recruitment	Volunteer participants were recruited from a local online forum for part-time jobs and flyers distributed across University of

Recruitment	Tsukuba. There are several conditions on recruitment to measure stable motor behavior to reduce the variance of data (See "Research sample" section above). Therefore, the effects reported in the present study might be overestimated if the variance of an entire population might be larger.
Ethics oversight	University of Tsukuba ethics committee

Note that full information on the approval of the study protocol must also be provided in the manuscript.
